# Host Sexual Dimorphism and Parasite Adaptation

**DOI:** 10.1371/journal.pbio.1001271

**Published:** 2012-02-28

**Authors:** David Duneau, Dieter Ebert

**Affiliations:** University of Basel, Zoological Institute, Basel, Switzerland

## Abstract

In species with separate sexes, parasite prevalence and disease expression is often different between males and females. This effect has mainly been attributed to sex differences in host traits, such as immune response. Here, we make the case for how properties of the parasites themselves can also matter. Specifically, we suggest that differences between host sexes in many different traits, such as morphology and hormone levels, can impose selection on parasites. This selection can eventually lead to parasite adaptations specific to the host sex more commonly encountered, or to differential expression of parasite traits depending on which host sex they find themselves in. Parasites adapted to the sex of the host in this way can contribute to differences between males and females in disease prevalence and expression. Considering those possibilities can help shed light on host–parasite interactions, and impact epidemiological and medical science.

## Abstract 2

Disease expression and prevalence often vary in the different sexes of the host. This is typically attributed to innate differences of the two sexes but specific adaptations by the parasite to one or other host sex may also contribute to these observations.

## Introduction

Males and females are sexually dimorphic because of divergent selection in many traits, including morphology, physiology, life history, and behavior. In fact, the most extreme differences described within species, such as body size, are often those between sexes and, typically, sex differences explain most of the phenotypic variation between adults in a sexual population. In populations of sexual species, parasite prevalence, disease symptoms, and virulence also often differ between males and females (see review in [1], recent examples in [2]–[5]). This effect of host sex, recorded even in humans, has mainly been attributed to sex-specific differences in immune response, hormones, and resource allocation [1],[6]–[11]. For example, the male hormone negatively affects the efficiency of the immune system. Other sex-dependent characteristics, however, including morphological, physiological, behavioral, dietary, and life history traits, may also contribute to these observations.

Parasite populations are expected to have adapted to the characteristics of their most common host type [12]. If a parasite population evolves mainly in one sex (e.g., those transmitted among extremely sex-biased host populations), sex-specific characteristics may impact how the parasite adapts to that host (Table 1). Therefore, without considering the sex of the host in which the parasite primarily evolved, it is difficult to disentangle whether sex-biased parasitism is the result of differences among hosts only, or if adaptation of parasites contributed to these characteristics as well.

**Table 1:** Examples of sexually dimorphic traits that might influence parasite evolution.

Sexually Dimorphic Traits	Implications for Parasites	Examples
Sex-specific tissue	- Parasite adaptation to the tissue only present in one host sex (e.g., ovarian parasites of fish [49] and testicular parasites of fish [50]).	- Primary sexual traits.
Sex-specific properties of tissue	- Parasite adaptation to the specific host properties of a tissue existing in both host sexes. This may results in specific parasite communities adapted to the sex-specific properties (e.g., different microbial community on hands of different sexes [68]).	- Different skin properties (e.g., men sweating more than women [69]).- Differences in diet with implication on digestive apparatus (e.g., American bison males eat relatively more C4 plants and females more C3 plants [40]).
Sex-specific need/metabolism	- Parasite adaptation to resources available in each sex.	- Males with wings and females wingless (e.g., Velvet ants [70] might have different physiology and different needs.- Differences in diet for different needs (e.g., male capucin monkeys eating more animals than females [41]).

Here, we argue that the sex of the host can impose selection on the parasite itself, which in turn will contribute to variation in disease prevalence and expression among male and female hosts. This hypothesis could be tested in systems where hosts and parasites can be used in experimental infections and where parasite isolates can be obtained from both host sexes. But, to our knowledge, such experiments have never been done, probably because it is assumed that the prevalence and severity of disease found in different host sexes are caused by the characteristics of the host alone. We propose that parasite adaptation to specific host sexes can lead to three different evolutionary outcomes for the parasite: 1) parasites that adapt differently to each sex, leading to dimorphism in the parasite population, here called “host sex–specific dimorphism”, 2) parasites that specialize on only one sex: “single sex specialization”, and 3) parasites with phenotypically plastic traits, whose expression is dependent on the sex of their host: “plastic sex-specific disease expression”.

**Figure 1:**
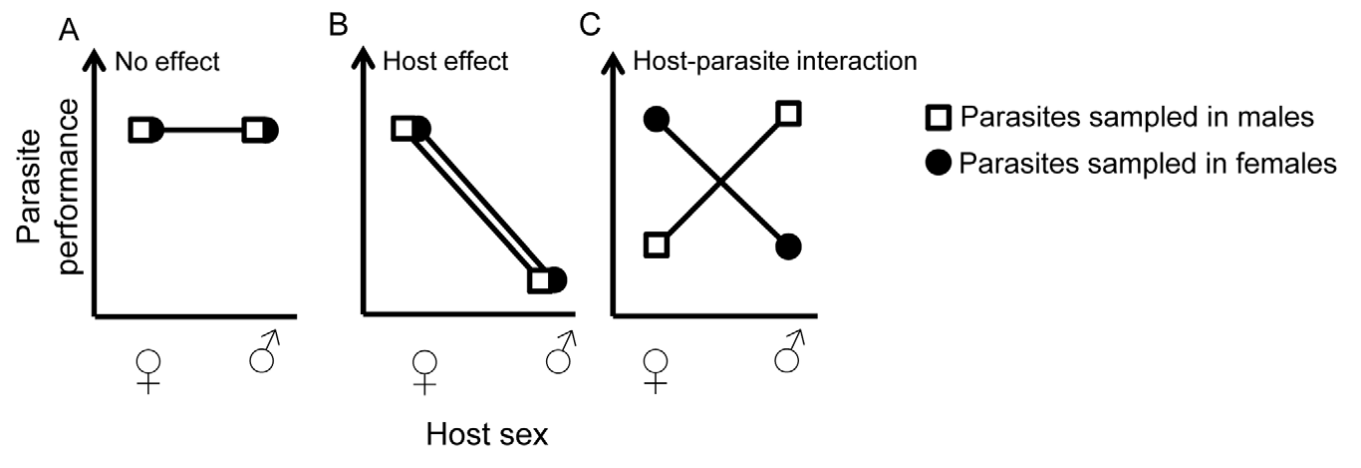
Possible outcomes of experimental tests with parasites sampled and tested in male and female hosts.

**Figure 2:**
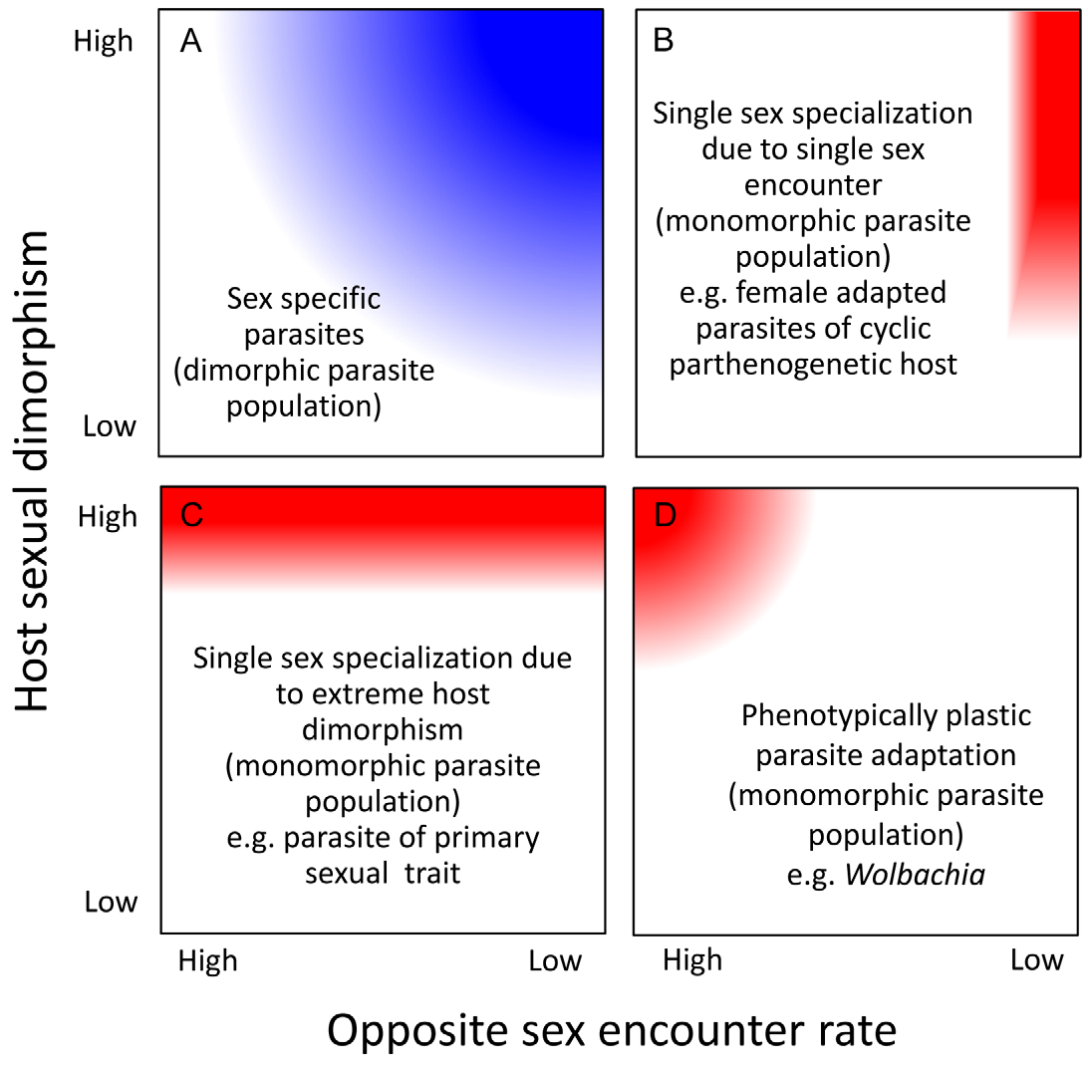
Parasite evolution in relation to host sexual dimorphism and likelihood of encountering the other host sex. In red and blue are parameter combinations, which lead to monomorphic or dimorphic parasite populations, respectively. The higher the degree of host sexual dimorphism and the lower the probability of encountering the same host sex, the higher the likelihood is that a parasite will adapt specifically to its common host sex (A). When one host is different from the other, and so rare that a parasite cannot persist in it (e.g., males in a facultative sexual species like many rotifers, cladocerans, and aphids), then the parasite species may specialize entirely on the common sex (B). When one host is very different from the other in a trait important for the parasite (e.g., a primary sexual trait), then, disregarding the rate at which the opposite sex is encountered, the parasite may specialize entirely on the more suitable host (C). When males and females are very different from the parasite's point of view and the parasite encounters both sexes equally often (D), the parasite might evolve phenotypic plasticity (e.g., *Wolbachia*).

We will begin by explaining in more detail these three evolutionary scenarios using a simple experimental design to help distinguish them (Box 1, Figure 1). The conditions under which these scenarios may evolve differ strongly. We also attempt to pinpoint those conditions that are likely to play a crucial role for the evolution of sex-specific parasite adaptation and lead either to monomorphic parasite populations or to dimorphic parasite populations (Figure 2). Then, we discuss how host demographic properties, notably host sex ratio and social structure, can influence the extent to which the parasite evolves. Specifically, differences between host sexes can affect the likelihood and extent of transmission of parasites and disease among host sexes and determine how they change the selective environments for the parasites. We conclude by considering the implications of host sex–specific adaptation for studies for ecology and evolutionary biology but also for applied subjects such as medicine, veterinary medicine, and agriculture. An explicit consideration of these possibilities will help us understand the commonly observed differences in the distribution of infectious diseases among different sexes.

**Box 1. How to Test Whether a Parasite Is Specifically Adapted to the Sex of Its Host**

There are many examples of differences in parasite prevalence and/or infection symptoms between male and female hosts. Such differences, however, are typically interpreted in terms of the characteristics of host individuals rather than that of the parasites. For example, there may be differences because the male or female host provides a more or less suitable environment for the parasite. Distinguishing this from a parasite that has traits that are specifically adapted to one sex is challenging. Here, we propose an experimental approach to highlight parasite adaptations to host sex. This follows the same type of design used to test for local adaptation in various ecological systems [14], but, instead of comparing distinct geographical populations, we compare parasite populations isolated from either male or female hosts.

Using a full factorial design, you can expose female and male hosts (“test environment”) to parasites sampled from either female or male hosts (“origin environment”), and measure parasite performance, via a variety of phenotypic traits. This can be done in any system where parasite harvest and infection are possible in both host sexes. The way parasite performance is measured will depend on the specific biological system and can include traits such as infectivity, virulence, survival, and production of parasite transmission stages. Figure 1 illustrates the three main types of possible outcomes. First, if parasite performance is not affected by the test or the origin environments (Figure 1A), you might conclude that parasites sampled in males and females do not correspond to divergent populations (or have not diverged for that specific measurement of performance). Parasites might either express the same traits in both host sexes (i.e., no sex-specific adaptation), or might have evolved traits that are expressed plastically, depending on which sex they infect (we refer to this as “plastic sex-specific disease expression” in the main text). Second, if parasite performance differs between test environments but not between origin environments (Figure 1B), it also indicates that the parasite populations sampled in males and females did not diverge. But in this case, the parasite is either specialized on one host sex (single-sex specialization), and/or one host sex is a more suitable “habitat” than the other. To distinguish between no sex-specific adaptation and plastic sex-specific disease expression in the first case, or between single-sex specialization and a sex bias in host suitability in the second, you would need to investigate parasitic traits that you suspected might represent specific adaptations. It would be necessary to determine whether and how these also differ in relation to the test and the origin environment. Finally, when parasite performance depends on a combination of test and origin environments (i.e., when there is an interaction effect between the two factors; Figure 1C) we can conclude that the parasites sampled in male and female hosts have diverged and are sex-specifically adapted.

In addition to these quantitative analyses of parasite phenotypic traits, a population genetic approach can provide further information. Population genetic methods can be used to estimate the extent of genetic divergence between parasites collected from male versus female hosts [79] and to find candidate loci under selection. Population genetic methods have been used, for example, to establish differences between HIV viral populations sampled in humans reporting on distinct subtypes associated with male homosexual versus heterosexual transmission [80].

## Three Paths to Adaptation

### Host Sex–Specific Dimorphism

Male and female hosts may represent very different environments to which parasites adapt specifically. This is analogous to local adaptation, where resident genotypes in a specific environment are, on average, fitter than genotypes originating from other environments [13]. Local adaptation implies antagonistic pleiotropy, whereby the selected alleles have opposite effects on fitness in different environments—in other words, there is a trade-off in performance between the environments [14]. One can equally view the two host sexes as two different environments. The trade-off is expected to result in parasite origin × host sex interactions for parasite fitness (Figure 1C). In that context, the evolution of parasite divergence in a sexual host depends mainly on two parameters, the extent to which the host is sexually dimorphic (difference between environments) and the likelihood of a parasite encountering the opposite sex—the alternative environment—during transmission (Figure 2). The latter is conceptually similar to gene flow between environments. If parasite populations are structured by host sex, the parasite populations may have the opportunity to adapt to the conditions specific to the host sex they encounter most often. Thus, the parasite would evolve a host sex–specific dimorphism (Figure 2A).

### Single Sex Specialization

We discuss here two ways by which a parasite may become adapted to one sex only. In extreme cases, one host sex may be so rare (e.g., males in cyclically parthenogenetic species, such as aphids, are absent for large parts of the year) that the parasite rarely encounters them (Figure 2B). In this case, parasites sampled from the rare host would actually be adapted to the other sex (the common sex), and parasites from both origins would be fitter in the common host sex (Figure 1B). Alternatively, the parasite could adapt to a host trait that is found in only one host sex, such as primary or secondary sexual traits. The parasite populations may adapt only to this sex, even if the likelihood of encountering the other sex is high (Figure 2C). In this case, parasites sampled in the host sex to which they are not adapted (if this is possible), would perform better in the opposite host sex (Figure 1B).

### Plastic Sex-Specific Disease Expression

Phenotypic plasticity, a property whereby the same genotype translates into distinct phenotypes depending on the environment, is a common way for organisms to deal with fluctuating environments [15]. Parasites facing distinct male and female host environments might have evolved plasticity in relation to those environments and be able to express host sex–specific traits accordingly. Following Scheiner [16], the plastic expression of a trait is favored when 1) variability among environments is high, 2) environments are equally abundant, 3) the strength of selection is equal in both environments, 4) the environmental cue determining the phenotype is highly correlated with the environment of selection, and 5) the cost of plasticity, which is the cost of maintaining the genetic and cellular machinery necessary to be plastic, is compensated by its advantage. If these conditions are met, phenotypic plasticity is expected to evolve (Figure 2D); otherwise, a single generalist phenotype will be favored. If there is plasticity, then parasites originating from different host sexes will be equally fit when tested in the same sex environment (Figure 1A).

## Host Population Structure and Parasite Transmission

The evolution of sex-specific parasite adaptation is affected by the likelihood of parasites being transmitted within or between host sexes (Figure 2). This depends strongly on the host species and the ecological circumstances (Table 2). Here, we focus mainly on cases where the likelihood of encountering a host of the opposite sex is low. For example, males and females are not always equally abundant and, therefore, parasite transmission will occur among the most common sex. Biased sex ratios are often observed in natural populations [17]–[20], and are even an intrinsic characteristic of certain species, for example, the abundance of females in cyclically parthenogenetic species (e.g., aphids, cladocera, rotifers), in sequential hermaphrodite species [21], and in many haplodiploid species such as ants, bees, wasps, and mites. Parasites infecting social bees, wasps, and ants will face mostly female workers and will only rarely encounter males. For bumble bees, it has been shown that foraging female workers are more infected by tracheal mites than foraging males [22]. Female-biased sex ratios can also result from sex-ratio distorters such as *Wolbachia* bacteria, which infect at least 20% of all insect species [23].

**Table 2:** Examples of host sex differences that might influence parasite evolution.

Host Sex Difference		Examples and Their Implications for Parasites
Exposure	Differences in visited areas	- Male spadefoot toads spend many nights in water while females go only once for a few hours, which results in males being the common hosts for aquatic parasites such *Pseudodiplorchis americanus* [71].- Cormorant males and females forage in different places, which results in parasites of one sex more likely to infect more this particular sex [36].
	Differences in behavior increasing parasite encounter risk	- Male mammals sniff urine and feces for establishment of social hierarchy, which results in increasing the contact with pathogens [33],[34] and male-to-male transmission.- House finch males prefer contact with less aggressive males while females have no preference, which results in increasing the likelihood of infection between males when the less aggressive males are more heavily infected [72].
	Differences in host availability	- Biased sex ratio in cyclically parthenogenetic species and in many haplodiploid species such as ants, bees, wasps, and mites may result in parasites more likely to infect only one host sex.
	Difference in social structures	- Spatial segregation of male and female hosts such as most ungulates, which results in the segregation of the parasite populations they carry.
	Differences in host body size	- Males are larger than females (e.g., mandrills, elephants, sea lions). Male *Bonellia viridis* (annelids) are drastically smaller than females (see dwarf males [31]). Strong sex size dimorphism increases the likelihood to encounter the larger host sex (e.g., mammals [28]).
Susceptibility	Differences in immunocompetence	- Interaction between endocrine and immune system [44]–[48], which results in males and females differing in ability to fight off parasites [43] and parasites having a greater opportunity to spread within male hosts (e.g., twice for striped plateau lizards [48]).
	“Haploid-susceptibility hypothesis”	- In haplodiploid species, females are diploid, males are haploid. The “haploid-susceptibility hypothesis” predicts that the haploid males are more susceptible [73] and might be the host type the most commonly successfully infected.
Development	Differences in lifespan	- Females living longer than males (e.g., male hymenoptera live for days, certain females for years [74], male marsupials of the species *Antechinus stuartii* die shortly after the breeding season, while females live for years [75]), which may result in more parasite generations within the same female host and a higher probability for female hosts to get infected during their lifetime.
	Differences in development	- In bees (*Apis cerana*), larvae development is longer in drones (males) compared to workers. *Varroa destructor* mites have a developmental time matching those of drones. Mites on worker host larvae cannot reproduce [76],[77]. *Varroa* mites can actively choose the drone brood cells [78].

In species where sex ratios are unbiased, social structures can lead to spatial segregation of males and females and, consequently, their parasites. Males and females may live in mixed social groups only for limited periods of their life cycle, such as those with a matriarchal social organization. In African elephants (*Loxodonta africana*), for example, mature males leave the group to be either solitary or to spend time with other males [24]. Sexual segregation is also common in ungulates ([25]; Table 2) such as the American bison, where bulls and cows are not in contact for 11 months of the year [26]. The purpose of such segregation may enable females to avoid contact with parasitized males [27], supporting our suggestion that parasite populations may remain isolated within a host sex.

## Host Sexual Dimorphism and Parasite Transmission

Sex-specific host traits may also affect the rate at which hosts of different sexes encounter parasites and vice versa (Table 2). For example, body size, which is often dimorphic, may be why parasites in mammals more often infect the generally larger males than females [28]. In many taxa, males are larger than females (e.g., many birds [29]), but the reverse is not rare in some groups (e.g., insects [30]) and can be extreme as is the case with dwarf males, such as barnacles [31],[32], potentially reversing or exaggerating the pattern of infection bias observed in mammals. Certain types of sex-biased behaviors are also linked to an increased risk of exposure to parasites. For example, in mice and other mammals, male-specific sniffing of urine and feces used to assess social hierarchy can increase contact with pathogens [33],[34]. In domestic cats, the feline immunodeficiency virus (FIV), a virus mainly transmitted via bites, occurs twice as much in males because of sex differences in their social behavior. Males also have a higher propensity to bite each other [35], opening up another potential route for increased transmission between males. Conversely, parasites associated with nests (e.g., fleas and ticks) will generally encounter mature females or juveniles (which, typically, have no pronounced sex differences) more often than they will encounter male hosts. Other sexually dimorphic behaviors that might explain differences in exposure to parasites (Table 2) include foraging (e.g., cormorants [36], squirrel monkeys [37], and blue-footed and brown boobies [38]), diet (e.g., Fore people's cannibalistic practices [39], the American bison [40], and capuchin monkeys [41]), and dispersal (reviewed in [42]). However, the effects of these differences on the evolution of parasites and on the likelihood of parasite adaptation to specific host sex remains to be explored.

Susceptibility of a host to parasite infection will depend on whether the parasite can overcome the host immune system and how well it can grow in the host. By affecting exposure and susceptibility, differences between male and female hosts in morphology and life history traits can influence the likelihood that a parasite encounters one or the other host sex and, therefore, the probability that it evolves host sex–specific adaptations (Figure 2). Differential susceptibility due to host immunity has been proposed many times in vertebrates and is attributed to the interaction between endocrine and immune systems [43]. Sex hormones also regulate innate and acquired immunity [44],[45], and, as mentioned at the outset, testosterone interacts with the immune system, presumably explaining the higher parasite susceptibility of male rodents [46],[47] and lizards [48]. Whether a parasite can infect a host also depends on host physiology and on the resources that the parasite can exploit. In extreme cases, where the parasite infects a primary or secondary sexual trait (e.g., fish ovary parasites [49] and fish testis parasites [50]), only one sex is a suitable host. Males and females also differ in the type and concentrations of hormones and metabolites (Tables 1 and 2) such as body fat, which can be an important resource for parasites. In insects, for example, the females are larger [30] and often have a higher proportion of body fat. Space and nutrition are key components of the host's carrying capacity for any parasite population and so will have an impact on the number of generations a parasite population can have within the same host individual. Longer host lifespan can also increase the number of possible parasite generations, which increases the opportunity that the parasite has to adapt to its host's characteristics [51]. Sex differences in lifespan are quite common and can be extreme (Table 2).

## Evidence for Parasite Sex-Specific Adaptation

The examples above suggest that male and female hosts can represent different selective environments, with distinct challenges but also different opportunities for parasite growth. In addition, parasites might not be equally likely to encounter both sexes and may even be genetically isolated within host sexes. That parasites have the potential to form two sub-populations adapted to the sexes they infect the most appears reasonable. However, there are very few documented examples of parasite adaptation to host sex, and to our knowledge no example of a host sex–specific dimorphism has been described. There are a few recent empirical tested examples, however, of parasites actively choosing to infect the sex they most commonly encounter and, where they have the highest fitness, these make a compelling case for single-sex specialization of parasites (Box 2). This scarcity of evidence in general may reflect a lack of studies where this has been explicitly investigated.

**Box 2. Examples of Parasites That Are Adapted to One Sex of the Host**

The idea that parasite populations can adapt to only one of the sexes of their host or diverge to adapt to both host sexes is novel, and sex differences in infection success and/or symptoms have not been interpreted (or analyzed) from this perspective. While, to our knowledge, no example of a host sex–specific dimorphism has been described as such, some known parasite adaptations may correspond to single-sex specializations or plastic sex-specific disease expression (as described in the main text). Here, we refer to some examples that illustrate different aspects of parasite adaptation to host sex. There are many parasites that exploit either exclusively or predominantly only one sex of their hosts. Some of these have evolved mechanisms for discriminating between the sexes, thus ensuring they only infect suitable individuals. Others have evolved mechanisms for manipulating the infected host so as to recover particular sex-specific traits necessary for parasite proliferation and/or transmission.

**Discriminating the Sex of the Host**

Myxozoa belonging to the genera *Kudoa* are myxosporean parasites of fish that comprise around 70 species [81], of which all but one infect multiple hosts tissues. The only exception is the species *Kudoa ovivora*, which specifically infects the host's ovaries [49]. Curiously, to our knowledge, this parasite is the only species of the genus that infects exclusively sequential hermaphrodites where fish develop first as female and then become male (e.g., labrids and scarids). Such fish populations are known to have female-biased sex ratios [21], which could explain that this parasite adapted specifically to the characteristics of female hosts. The relevance of host sex ratio for parasite single-sex specialization is further discussed in the main text.

**Figure 3:**
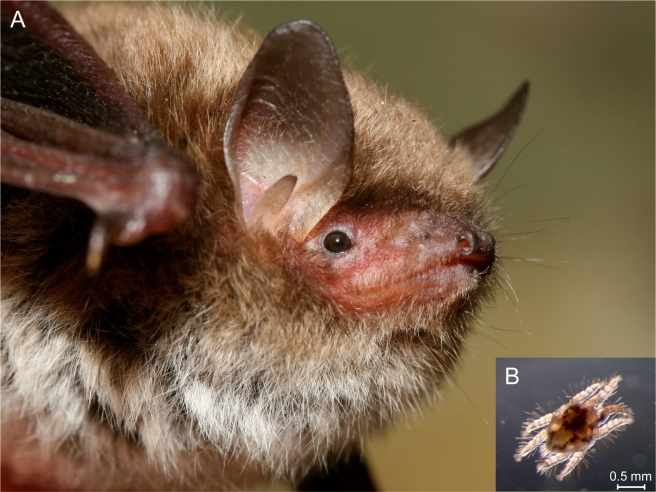
Photos of the ectoparasitic mite *Spinturnix andegavinu*s (B) and of its host bat *Myotis daubentoni* (A) to which the parasite is sex specifically adapted. Image credit: Manuel Ruedi and Philippe Christe.

The ectoparasitic mite *Spinturnix andegavinus* (Figure 3B) is mainly transmitted among “maternity clusters” of its host, the bat *Myotis daubentoni* (Figure 3A). Experimental studies have shown that these mites are capable of growing only on female hosts [82], which necessarily means that they are specifically adapted to this host type. The same studies also revealed that the parasite actively chooses to attach to females [82], and that selection for being on the correct host was sufficiently strong to favor mechanisms (possibly via sense organs) for the parasite to discriminate between host sexes. Many endo- and ectoparasites are known to be able to actively choose between host species [83]–[88], and even between host individuals of the same species [89]. It might be possible that host sex discrimination is more widespread than is commonly believed.

The mite *Varroa destructor*, an ectoparasite of bees and a great problem in apiculture, has a life cycle that includes a phase on adult bees, where the parasite spreads, and a phase on the developing host individuals inside the brood cells, where it reproduces [90]. In its original host, the Eastern honey bee *Apis cerana*, the mite reproduces exclusively in the presumptive drone (male bee) cells [76],[77],[91]. Mites carried into the brood cells by the adult nursing workers will stay in the brood cell if the larva within that cell is a presumptive drone, but not if it is a developing worker or queen (being repelled by a substance in the royal jelly fed to these larvae [92]). Brood cells with worker larvae are typically much less frequently visited by nursing adults [93], and this might have been the original trigger of the sex bias in parasite infection. In the more recent host *Apis mellifera*, where the parasite can reproduce in both drone and worker larvae, the difference in nurse care can partly explain that drone cells are around 10-fold more infected than worker cells [93],[94].

**Manipulating the Sex of the Host**

When a parasite is highly specialized on the characteristics of one host sex, infection of the “wrong” host type can carry high fitness costs; for example, if one sex-specific aspect of host anatomy is necessary for parasite growth or transmission. For sex-specialized parasites exposed to both host sexes, the cost of infecting the less suitable host type might be overcome by either a plastic response (i.e., the parasite will express different traits in different host types) or the manipulation of the host (i.e., the parasite will manipulate the traits of the host of the “wrong” sex). Host-sex manipulation has been described, for example, for parasitic barnacles of the genus *Sacculina*, which infect and sterilize crabs [95]. The parasite grows in the place where the host eggs are incubated (i.e., underside of the rear thorax), and spreads when female hosts perform egg-laying behavior. When these parasites infect male crabs, they induce the feminization of both morphology and behavior of infected males and, as a consequence, the parasites can be transmitted. The mechanism by which this feminization is induced is not well understood, but presumably involves the secretion of hormones by parasites [96]. If this secretion occurs inside male hosts but not inside female hosts, one can talk about plasticity in parasite traits relative to host sex. If, on the other hand, the secretion occurs in both infected females and males, one can talk about single-sex specialization of the parasite in the sense that this parasitic trait is adaptive only in males.

A typical example of phenotypically plastic response to host sex is that of bacteria from the large group of the Rickettsia (e.g., *Wolbachia* [97]) and sex ratio–distorting Microsporidia [98]. These parasites are well known and widespread examples of maternally transmitted parasites that are sex-specifically adapted. These endosymbionts are transmitted transovarially to male and female progeny, but have different behaviors depending on the host sex they infect. For example, *Wolbachia* may induce feminization of genetically male hosts or specifically kill infected males to favor infected females of the same brood [97]. *Wolbachia* is widespread in insects and is a compelling illustration of the importance of sex-specific parasite adaptations. It is likely that many other cytoplasmic parasites show sex-specific adaptations to increase their transmission.

### Implications of Parasite Sex-Specific Adaptation

Host sex–specific parasite divergence has implications for both host and parasite populations and for the dynamics of the interactions between them. Between-sex differences can represent a challenge for parasites, making it difficult to fully adapt to both sexes in well mixed populations. There are occasions when parasites encounter a high proportion of the host sex that they are not adapted to. For example, in organisms with cyclical parthenogenesis (e.g., *Daphnia*, aphids, rotifers), males may be absent for most of the time but common during a particular period of the year and/or under certain environmental conditions. Likewise, in many ungulates, males and females that live apart come together during the breeding season. This can have evolutionary consequences for the parasites that could either reduce or reinforce the adaptation. For the former, sex-specific adaptations may decrease or be eliminated when generalist parasites are favored over sex-adapted ones. However, reinforcement may occur if there is selection for parasite traits that enable the parasites to discriminate between host sexes, and thus help avoid the wrong host type (e.g., active host choice), or, in invertebrates, sex manipulations (e.g., feminization of male crabs by *Sacculina*, see Box 2). Finally, encountering the “wrong” host may also lead to the expression of unintentional disease symptoms that are actually detrimental to the parasite [52]. In extreme situations, parasite populations adapted to one or the other host sex might eventually become isolated from each other (dimorphic parasite population, Figure 2) and form different parasite species, each specialized on one host sex (monomorphic parasite population, Figure 2B and 2C).

Parasite sex-specific adaptations and the possibility for host sex change may be exploited by the host itself. For example, in the sequentially hermaphroditic fish *Thalassoma bifasciatum*, when the hosts are females they can be infected with the parasite *Kudoa ovivora*, which is specialized on exploiting only the host ovaries (Figure 2C). Interestingly, when infected, the hosts are able to change sex, removing the only resource the parasite can exploit and bringing it to a dead end [53].

Parasite adaptation to host sex can have important implications for host–parasite coevolution. We have proposed that the sex of the host can drive parasite sex-specific adaptation when parasite subpopulations evolve mainly in one host sex. For the host, however, selection on one sex only can be impaired by intra-locus sexual conflict [54],[55] when alleles that confer parasite resistance or tolerance in the affected sex decrease fitness of the other sex. The expression of traits associated with parasite resistance may thus become sex limited.

Host sex–specific adaptation of one parasite might also lead to sex-specific adaptation of other associated parasites. This may be the case, for example, for endoparasites transmitted by host sex–biased ectoparasitic vectors. In Box 2, we list examples of ectoparasites infecting predominantly or exclusively one host sex (e.g., the mites *Spinturnix andegavinus* that infect female bats of the species *Myotis daubentoni*). Such ectoparasites are likely to be vectors of different endoparasites, and, if the vector reproduces exclusively in one host sex, the vector-borne pathogens will also more often infect that host sex and may be selected in that environment.

Host sex is a key factor in studies in medicine and disease control and parasite sex-specific adaptation is a strong argument that both sexes need to be included equally in clinical trials, currently an important concern in medicine [56]–[60]. In humans, there are well documented host sex differences in parasite prevalence and infection symptoms, as well as prevention and treatment of infection. The immune system of men and women reacts differently to vaccines [61]. This difference can be vaccine strain–specific (e.g., men exhibited a higher antibody response than women for yellow fever vaccines from two of three different virus strains [62]). While this is undoubtedly related to intrinsic differences between men and women, if parasites then behave differently in male versus female hosts, either because of genetic divergence related to sex adaptation or because of phenotypic plasticity, then parasites in females and males might not be targeted by the same antibodies/drugs. Whatever the cause, failure to immunize/cure one fraction of the host population might create a reservoir for the parasites, and immunizing/curing one or the other sex can also have distinct effects on disease prevalence. Studies on the yellow-necked mouse show that treatment of male hosts reduced parasite prevalence in both sexes, but treatment of females reduced parasite prevalence only in females [63]. Even in the absence of sex-biased infection, there is a disproportionate contribution of male yellow-necked mice to parasite transmission [64].

### Prospects

Different types of host heterogeneity affect the evolution of infectious diseases [65]–[67]. Here, we have argued that the sex of the host is likely to be another important factor in parasite evolution. Documented host-sex differences in parasite prevalence or effect (see [1]) support the idea that the probability that parasites spread (within and between hosts) is not always the same with regard to host sex. These differences are generally attributed to intrinsic characteristics of the host individuals [1],[6]–[9]. The observed sex-biased disease prevalence and/or severity might indeed be due to the host's intrinsic heterogeneity, but might also be the result of the parasite having adapted to infect and grow in specific host sexes. Unequal host susceptibility and sex-specific adaptation by the parasite are not mutually exclusive explanations for sex-biased prevalence, and, in fact, must work together. The likelihood and extent of adaptation to a specific sex depends on many factors. These include characteristics of the host populations or host individuals that determine how different the male and female environments are, and how often the parasite experiences them. We discussed examples of each of these to illustrate how they can impact parasite evolution and lead to the divergence and specialization of parasite populations in different host sexes. Parasite characteristics, particularly the mode of transmission, will also have an impact on the likelihood of divergence between parasite populations in male and female hosts. Therefore, transmission mechanisms will affect sex-specific adaptation. For example, sexually transmitted parasites will typically have to deal with both host sexes and are less likely to adapt to any sex (represented by the left hand side of the *x*-axis in the Figure 2). Maternally transmitted parasites will be more likely to be adapted to females. To conclude, the sex bias of disease prevalence and severity is of a major current concern in parasitological studies, notably in medical trials [56]–[60]. We propose that by taking the possibility of parasite adaptation that is specific to the sex of the host into account, we will gain a better understanding of host–parasite dynamics and thus the possibility of parasite control and more generally of sex-related disease expression.
